# Sulfonated Pentablock Copolymer Membranes and Graphene Oxide Addition for Efficient Removal of Metal Ions from Water

**DOI:** 10.3390/nano10061157

**Published:** 2020-06-12

**Authors:** Simona Filice, Marta Mazurkiewicz-Pawlicka, Artur Malolepszy, Leszek Stobinski, Ryszard Kwiatkowski, Anna Boczkowska, Leon Gradon, Silvia Scalese

**Affiliations:** 1 Istituto per la Microelettronica e Microsistemi, Consiglio Nazionale delle Ricerche (CNR-IMM), Ottava Strada n.5, I-95121 Catania, Italy; 2 Faculty of Chemical and Process Engineering, Warsaw University of Technology, ul. Warynskiego 1, 00-645 Warsaw, Poland; Marta.pawlicka@pw.edu.pl (M.M.-P.); artur.malolepszy@pw.edu.pl (A.M.); lstob50@hotmail.com (L.S.); leon.gradon@pw.edu.pl (L.G.); 3 NANOMATERIALS LS, Wyszogrodzka 14/38, 03-337 Warsaw, Poland; 4 Institute of Textile Engineering and Polymer Materials, University of Bielsko-Biała, Willowa 2, 43-309 Bielsko-Biała, Poland; rkwiatkowski@ath.bielsko.pl; 5 Faculty of Materials Science and Engineering, Warsaw University of Technology, ul. Woloska 141, 02-507 Warsaw, Poland; anna.boczkowska@pw.edu.pl

**Keywords:** graphene oxide, sulfonated pentablock copolymer, metal ions, adsorption, water purification

## Abstract

Nowadays heavy metals are among the higher environmental priority pollutants, therefore, the identification of new, effective, reusable and easy-to-handle adsorbent materials able to remove metal ions from water is highly desired. To this aim, in this work for the first time, sulfonated pentablock copolymer (s-PBC, Nexar™) membranes and s-PBC/graphene oxide (GO) nanocomposite membranes were investigated for the removal of heavy metals from water. Membranes were prepared by drop casting and their chemical, structural and morphological properties were characterized using scanning electron microscopy (SEM), Fourier transform infrared (FT-IR) spectroscopy, dynamic mechanical analysis (DMA) and small-angle X-ray scattering (SAXS). The adsorption abilities and adsorption kinetics of both the polymer and the s-PBC/GO nanocomposite were investigated for the removal of different heavy metal ions (Ni^2+^, Co^2+^, Cr^3+^ and Pb^2+^) from aqueous solutions containing the corresponding metal salts at different concentrations. The investigated s-PBC membrane shows a good efficiency, due to the presence of sulfonic groups that play a fundamental role in the adsorption process of metal ions. Its performance is further enhanced by embedding a very low amount of GO in the polymer allowing an increase by at least three times of the adsorption efficiencies of the polymer itself. This can be ascribed to the higher porosity, higher roughness and higher lamellar distances introduced by GO in the s-PBC membrane, as evidenced by the SEM and SAXS analysis. Both the polymeric materials showed the best performance in removing Pb^2+^ ions.

## 1. Introduction

Over the next few years, within the high rate of population growth, the demand for fresh water is expected to increase, but its quality is seriously limited by anthropogenic activities: around two million tons of waste are produced by humans and disposed every day in water courses. Organic contaminants are present in water due to the decomposition of living materials and anthropogenic activities [1]. In particular, the mass use of cosmetic, cleaning and pharmaceutical products increases the level and variety of water pollutants, which are not removed by conventional wastewater treatment. Usually these contaminants are removed by coagulation followed by an advanced treatment process, such as membrane filtration, ion exchange/adsorption and ozonation/biodegradation. An overarching goal for providing safe water is to disinfect water from traditional and emerging pathogens, without creating more problems due to the release of toxic by-products [2]. Therefore, innovative approaches that enhance the reliability and robustness of water decontamination are needed. In addition to the above mentioned contaminants, most surface and ground water sources are contaminated with many heavy and/or radioactive metals due to anthropogenic sources (metallurgical, mining, chemical manufacturing and battery manufacturing industries) or geological reasons [3,4]. Although metals and metalloids play significant roles at trace levels in living organisms, at higher concentration levels these are toxic and/or carcinogenic. If it exceeds its critical level, Ni is not only carcinogen like Cr, but it might also cause gastrointestinal distress, pulmonary fibrosis and skin dermatitis [5]. Co has harmful effects such as acute and chronic disorders or genetic mutations [6]. The International Agency for Research on Cancer (IARC) has classified Cr(VI) as a group I carcinogen. Cr(VI) compounds can enter cells via the sulfate-anion channel and then be subjected to reduction by a wide variety of cellular reductants. During this process, molecular oxygen is activated and a wide spectrum of reactive oxygen species (ROS) is generated. Consequently, excessive ROS may give rise to oxidative stress and DNA damage [7]. Pb is the most important toxic heavy element in the environment [8]: due to its non-biodegradable nature and continuous use, its concentration accumulates in the environment with increasing hazards. In particular, blood disorders and damage to the nervous system have a high occurrence in Pb toxicity.

Thereby, it is necessary to eliminate the toxic heavy metal ions from wastewater preventing their release into the environment. Traditional techniques for the elimination of heavy metal ions include precipitation, membrane filtration, sorption and ion exchange [9]. Chemical precipitation has been traditionally used for heavy metals removal due to its simplicity, but this method is ineffective for low metal concentrations and it generates large amounts of sludge to be treated. Secondary pollution is also the main disadvantage of the use of ion-exchange resins for heavy metals removal from wastewater. One of the most suitable methods for heavy metals removal is adsorption; in this case the efficiency of the removal process depends on the characteristics of the adsorbents, in particular they must have a high surface area and must be selective, low cost, non-toxic and regenerable.

In recent years, the use of nanomaterials and polymeric nanocomposites in water purification has been shown to be very promising, since it allows multiple approaches to be combined, such as photocatalysis, adsorption and membrane technology; furthermore, it can provide the most appropriate solutions according to the specific problem and the natural and economic resources of developing countries [10,11].

Among nanomaterials, graphene-based materials have been widely investigated in recent decades for the removal and detection of environmental pollutants due to their unique physicochemical properties [12]. In particular, graphene oxide (GO) combines the advantages of a two-dimensional structure with a high surface area, enhanced active sites and abundant functional groups on its surface [13]. GO can be easily obtained via an oxidation process starting from graphite and resulting in single or a few graphene-like layers with oxygen functional groups (i.e., epoxy, carboxyl, carbonyl, hydroxyl, etc.) on their surface. These groups confer to graphene-like layers high hydrophilicity and the possibility of being functionalized, resulting in a higher selective adsorbent for water purification. GO was selectively modified using laser irradiation [2] or chemical modifications [14] to confer its antimicrobial properties and higher adsorption selectivity and capacity for dye removal from water.

All these characteristics make graphene oxide a good candidate for the pre-concentration and recovery of heavy metal ions from large volumes of aqueous solutions. Zheng et al. [15] reported low-temperature exfoliated graphene nanosheets (GNS), which could be used to adsorb Pb^2+^ from aqueous systems. According to Zhao’s group [13], a few layered GO nanosheets also had a higher adsorption capacity for Cd^2+^ (0.106 g g^−1^) and Co^2+^ (0.068 g g^−1^). In an earlier study [16], the affinity between cations and GO was investigated by combining adsorption measurements with aggregation kinetics: metals valence, electronegativity and hydration shell thickness mainly affect their affinity with GO sheets, following the order of Cr^3+^ ≫ Pb^2+^ > Cu^2+^ > Cd^2+^ > Ca^2+^ > Mg^2+^ ≫ Ag^+^ > K^+^ > Na^+^.

Although nanoscale materials such as GO show considerable improvement in terms of their physical and chemical properties, their size remains the main problem in a large-scale water treatment process because of the need for post-treatment recovery, which is necessary to avoid their impact on the environment and human health. GO is only one atom thick, with a lateral length of several hundred nanometers making it highly stackable and self-assemblable; laminar GO membranes are a promising candidate to develop nanofiltration membranes with an ultrahigh permeability [17]. Anyway, until now, GO membranes suffer from low stability in water due to the high hydrophilicity of GO; thus, the GO membrane design and self-assembly technique has to be improved to enhance its stability under various conditions. On the contrary, GO composite membranes obtained by immobilizing GO flakes inside a polymeric matrix have been shown to be a promising approach for water purification [10,11,18]. In this regard, polymers seem to be very promising immobilizing substrates due to several advantages such as flexible nature, low-cost, chemical resistance, mechanical stability, low density, high durability and ease of availability. This approach allows reusable and easy-to-handle adsorbents for water purification, and these nanocomposites can also be investigated in membrane filtration technologies, showing great potentialities for heavy metal removal. The adsorption of heavy metals occurs as a result of physical or chemical interactions between the functional groups on the surface of the adsorbent and the aqueous contaminants, according to the strength of the interaction of the heavy metals/functional groups. Many synthesized polymers or biopolymers with special functional groups (e.g., amine, carboxyl and sulfonic acid) also show efficient adsorption capacity for heavy metal ions [19]: Cr(VI), Zn(II), and Pb(II) can be removed from wastewater by polypyrrole based adsorbents and quaternized polysulfone and polystyrene ethylene butylene polystyrene (PSEBS) membranes can be used as adsorbents. Another sulfonated polymer, i.e., Nafion 117 was investigated by Nasef and Yahaya [20] for the removal of Ni(II), Co(II), Pb(II), Cu(II) and Ag(I) metal ions via adsorption up to five cycles.

In this work, sulfonated pentablock copolymer (s-PBC) membranes and hybrid nanocomposite s-PBC/GO membranes were investigated for the first time for the removal of heavy metals from water.

S-PBC is a sulfonated pentablock copolymer (s-PBC: tert-butyl styrene, hydrogenated isoprene, sulfonated styrene, hydrogenated isoprene, tert-butyl styrene (tBS-HI-SS-HI-tBS)), commercialized by Kraton Performance Polymers Inc. (Houston, TX, USA) as Nexar™. The advantages of this polymer are mainly low cost, good processability, ease of functionalization, and the possibility to combine different properties owned by the hydrophobic and hydrophilic domains. The molecularly designed Nexar™ structure results in a polymer with controlled swelling and good mechanical properties in the hydrated state. In addition, this type of precursor has a micellar structure that can be modulated at the polymeric level with a formation of preferential channels for filtration application [18]. Due to all these properties, recently, Nexar™ has been investigated in water purification as an adsorbent or for the immobilization of inorganic photocatalysts [18,21], in desalination applications [22] and as a proton exchange membrane in water electrolysis [23].

The aim of this work is to present for the first time the use of Nexar™ as adsorbent for heavy metals removal and to further improve its performance by adding GO. Hybrid polymeric membranes were prepared by dispersing graphene oxide in s-PBC. Both the s-PBC and the hybrid s-PBC/GO membranes have been tested as adsorbent media for Co^2+^, Ni^2+^, Pb^2+^ and Cr^3+^ ions removal from water. The good adsorption abilities of the polymeric membrane, in terms of total amount of adsorbed ions and removal rate, were further improved by the presence of a very low amount of GO, which is embedded in the polymeric matrix and, therefore, is not released in the environment.

## 2. Materials and Methods

### 2.1. Materials

**Figure 1:**
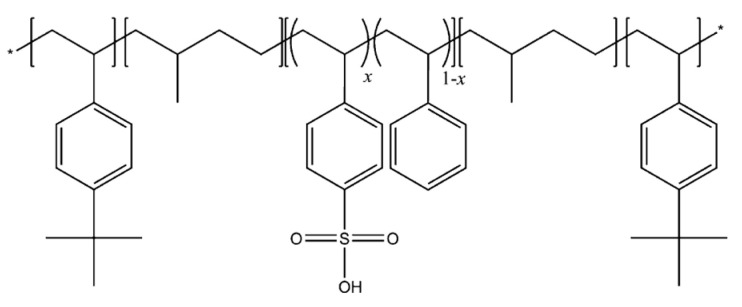
Molecular structure of sulfonated pentablock copolymer (s-PBC) (Nexar™).

A sulfonated pentablock copolymer poly(*t*BS–HI–sS:S–HI–*t*BS) solution, or s-PBC, with 10–12 wt% polymer in a cyclohexane/heptane mixed solvent was provided by courtesy of Kraton Polymers LLC. A scheme of this copolymer, commercially available as Nexar™, is reported in Figure 1. The ion exchange capacity (IEC) value of the commercial polymer is 2.0 meq. g^−1^ corresponding to a sulfonation degree of 52 mol%. The molecular weight is 112,500 g mol^−1^ and the volume fraction (*t*BS–[sS:S]–HI) is 0.300–[0.226:0.208]–0.266 [23].

Graphene oxide was prepared via a modified Hummers method [24,25]. Generally, for this process 10 g of graphite (Acros Organics, Thermo Fisher Scientific, Geel, Belgium), 4.7 g of sodium nitrate, 30 g of potassium permanganate and 230 mL of sulfuric acid (98%) were used. The obtained suspension was kept in an ice bath to keep the temperature below 10 °C. Then, the mixture was heated up to 30 °C and kept under stirring for 2 h. In the next step, 100 mL of water was added, which resulted in an increase of the mixture temperature up to 100 °C. Finally, the solution was treated with 10 mL of 30% H_2_O_2_ and kept in an ultrasonic bath for 1 h. For purification, the slurry was washed using filtration with deionized water until the pH of the filtrate reached ~7. All the chemicals were analytical grade, purchased from Chempur (Piekary Śląskie, Poland).

The salts, Co (II) chloride CoCl_2_ ∙ 6H_2_O (98%, p.a.) and Ni (II) nitrate Ni(NO_3_)_2_ ∙ 6H_2_O (99%, p.a.), were purchased from Carl Roth (Karlsruhe, Germany). Cr (III) sulfate ∙ 18H_2_O (100%, p.a.) and Pb (II) nitrate (98%, p.a.) were purchased from Chempur (Piekary Śląskie, Poland). All the salt solutions were prepared by dissolving a proper amount of salt in deionized water.

Dimethylformamide (DMF, 99%, p.a.), used for preparation of the polymer and composite membranes, was purchased from Chempur (Piekary Śląskie, Poland) and was used without further purification.

### 2.2. Membrane Preparation

s-PBC membranes and hybrid s-PBC/GO nanocomposite membranes were prepared using the solvent casting method. In particular, a 0.3 g/mL polymer solution in DMF was prepared by drying 27 g of commercial s-PBC solution at about 60 °C to get the evaporation of the commercial solvents and then dissolving it in 90 mL of DMF (as revealed by the clear colour of the solution). Finally, 15 mL of the suspension were cast on a Petri dish and left overnight at 60 °C for the complete evaporation of the solvent. In the case of nanocomposites, 0.045 g of GO aqueous solution (1.2 %wt) was added slowly to 60 mL of the polymer solution in DMF, prepared as described above. This final solution was stirred for several hours to get a homogeneous mixture until the solution was dense enough to be cast as described for the s-PBC membrane. Afterwards, the membranes were removed from the Petri dish by dipping the glass plate in deionized water for several minutes. Finally, they were pressed between two Teflon plates and heated in an oven at ambient condition at 150 °C for about 25 min. The membranes were soaked and washed in deionized water produced by a Millipore Advantage A10 system (Merck Millipore, Darmstadt, Germany) at room temperature in order to remove the eventual impurities, such as residual acids, until the soaking solution stabilized at neutral pH [18].

### 2.3. Characterization

Morphology and chemical mapping of the samples was performed using a field emission scanning electron microscope (Zeiss Supra 35 FE-SEM by Zeiss, Oberkochen, Germany) equipped with an EDX microanalysis system (X-MAX, 80 mm^2^ by Oxford Instruments, Abingdon, UK). In order to analyse the cross section of the membranes, they were dipped in liquid nitrogen, where they become stiff, and then they can be easily broken in two parts. IR analysis was performed using Nicolet iS10 (Thermo Scientific, Waltham, MA USA) FT-IR spectrometer equipped with an attenuated total reflectance (ATR) module with a diamond crystal. The samples in dry state were measured as prepared. The samples in wet state were measured after soaking the samples in water. The measurements were performed in 400–4000 cm^−1^ wavenumber range.

Dynamic mechanical analysis (DMA) analysis was performed using a DMA Q-800 TA Instruments Tester in the multi-frequency strain module under 1 Hz frequency, 0.1 amplitude strain, 0.05 N static force and 125.0% force track. The samples were in the form of 2 × 10 mm thin film. The test was performed in the range of temperature from −90 °C up to 180 °C with a 2.00 °C/min heating rate.

Small-angle X-ray scattering (SAXS) measurements were carried out with a compact Kratky camera equipped with the SWAXS optical system (Hecus-MBraun, Graz, Austria) and linear position sensitive counters PSD-50m (Hecus-MBraun, Graz, Austria). The Cu target Roentgen tube was used as the X-ray source (λ = 0.1542 nm), controlled by a PW10 high voltage generator (Philips Co., Eindhoven, Netherlands) operated at 40 kV and 30 mA. The samples investigated were in the form of a suitable sector of the membranes and they were fixed inside the sample holder. The slit-smeared SAXS patterns collected were analysed using the magnitude of the scattering vector: s = 2 sinθ/λ as the abscissa (where 2θ is the scattering angle and λ is the radiation wavelength). The SAXS patterns of the membranes were determined in the range of the s vector magnitude from 0.01125 nm^−1^ to 0.80 nm^−1^ (i.e., in the range of the 2θ scattering angle from ca. 0.10° to 7.05°).

The adsorption properties of s-PBC and GO/s-PBC were investigated by immersing a circular piece of membrane (2.5 cm of diameter) in 20 mL of salt solution. Each hour, 2 mL of each solution were taken for measurement. The amount of adsorbed and residual metal concentrations in the solution was measured using energy dispersive X-ray fluorescence (EDXRF) using an Epsilon 3XLE spectrometer (Malvern Panalytical) with Omnian standardless analysis solution software.

The pH value of the solutions was measured using litmus paper.

The water uptake value of each membrane was calculated in the following way, using a microbalance [18,23]:

$$\mathrm{Uptake}\%=\frac{\left(m_{wet}-m_{dry}\right)}{m_{dry}}\times 100$$

where *m_dry_* is the mass of the membrane dried in the oven at 60 °C for 2 h and then put in a desiccator, and *m_wet_* is the weight of the membrane after soaking in distilled water at room temperature for 48 h and quickly wiped with a paper tissue in order to remove most of the free surface water. The calculated water uptake values are 194% and 237% for s-PBC and s-PBC/GO, respectively.

## 3. Results and Discussion

### 3.1. Membrane Characterization

The morphology and the thickness of s-PBC and s-PBC-GO were investigated using SEM analysis.

**Figure 2:**
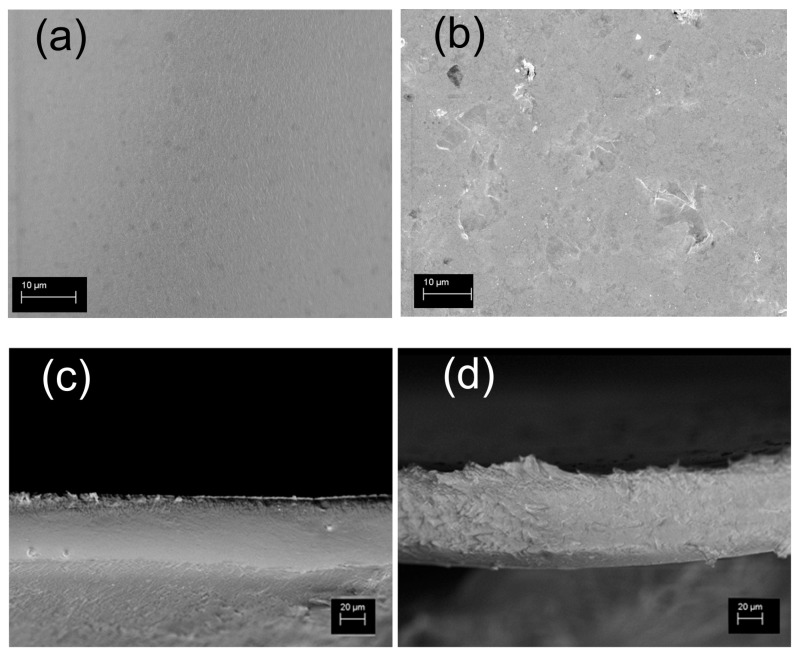
SEM images in plan view and cross section of s-PBC (**a,c**) and s-PBC/GO (**b,d**) membranes.

In Figure 2a–d we report the SEM images of the s-PBC membrane in plan (a) and tilted view (c) and the s-PBC/GO membrane in plan (b) and cross view (d). The s-PBC membrane is quite smooth and homogeneous, while SEM analysis of s-PBC/GO shows the presence of some graphitic planes on the surface (b) and confirms the complete and homogeneous dispersion of the filler through the entire volume of the polymeric matrix (d), forming a spongy structure with well visible graphitic planes. The thickness of s-PBC and s-PBC/GO membrane is 55 and 70 m, respectively.

**Figure 3:**
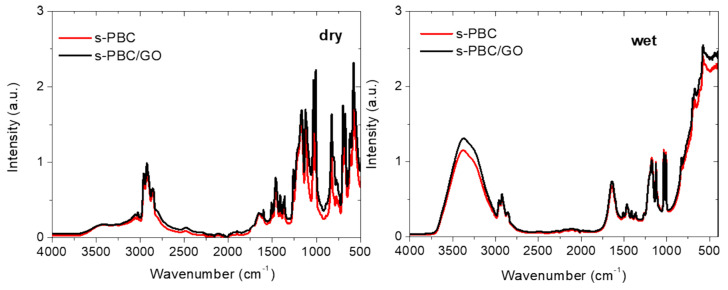
FT-IR spectra of s-PBC and s-PBC/GO nanocomposite acquired in dry (on the left) and wet (on the right) state.

FT-IR analysis of the polymer and the nanocomposite was performed in order to verify if the preparation of this membrane in a different solvent (DMF) with respect to the commercial product (prepared in apolar solvents) could affect its chemical structure. The spectra, reported in Figure 3, were acquired for the materials in the dry and wet state and these are normalized to the peak at 2925 cm^−1^. FT-IR spectrum of graphene oxide is reported in Figure S1 as a comparison.

For the s-PBC polymer, the peaks can be explained according to the assignment reported in [24], showing no difference (no chemical modification) with respect to the commercial material. We observe a peak at 1007 cm^−1^ due to the CH stretch of sulfonated polystyrene segment and the signals at 1033 and 1124 cm^−1^ resulting from the symmetric and antisymmetric stretching vibration of SO_3_H groups, respectively. In particular, the peak at 1124 cm^−1^ was demonstrated to be dependent on the number of sulfonic groups [18,23]. The absorption peaks at 1169 and 1200 cm^−1^ of s-PBC can be attributed to asymmetric stretching of SO_2_ in the sulfonic groups. Furthermore, three other absorption peaks at 2858, 2924 and 2958 cm^−1^ are observed and ascribed to asymmetric and symmetric CH stretching [26,27]. The peaks at 1645 cm^−1^ and in the region between 3000 and 3600 cm^−1^, related to water molecules inside the polymer, are less intense but still evident for the materials in the dry state, showing their high affinity for water [28]. If we observe the spectra of the s-PBC and s-PBC/GO in dry state, all the signals up to 1200 cm^−1^ are more intense for the nanocomposite since these are the sum of both the contributions of the polymer and GO. The characteristic signals coming from graphene oxide (see Figure S1) in the composite are not clearly visible due to the fact that there is a very small amount of GO added as a filler. The FT-IR measurements show that graphene oxide has a lot of functional groups present in its structure. Possible group assignment can be found in [24,29]. Indeed, IR absorbance features associated with the chemical functionalities of GO on both the edges and the basal plane, with an overlap of different derivatives of ethers (C-O), epoxides (C-O-C), aromatic C-H and C-OH phenols, appear in the region 800–1330 cm^−1^ [30]. The presence of phenolic species suggests a large amount of edges in the GO sheets, since phenols are positioned mainly on the edges of GO sheets. Generally, GO is rich in -OH bonds (which can be observed from the 3500 cm^−1^ and 1080 cm^−1^ absorption peaks) coming from water and hydroxyl groups, C-O-C groups observed from the ~1200 cm^−1^ peak and C=O bonds coming from carboxyl groups present in the peak at ~1700 cm^−1^. The stretching mode of sp^2^-hybridized C=C of GO is observed in the range of 1500–1600 cm^−1^ [30].

In the wet state (see Figure 3 on the right), the peaks at 1645 cm^−1^ and the region 3000–3600 cm^−1^ are due to hydroxyl and water contributions; these are higher for the nanocomposite with respect to s-PBC, underlying their higher water uptake with respect to s-PBC itself. This is in agreement with the water uptake values reported in the previous paragraph.

**Figure 4:**
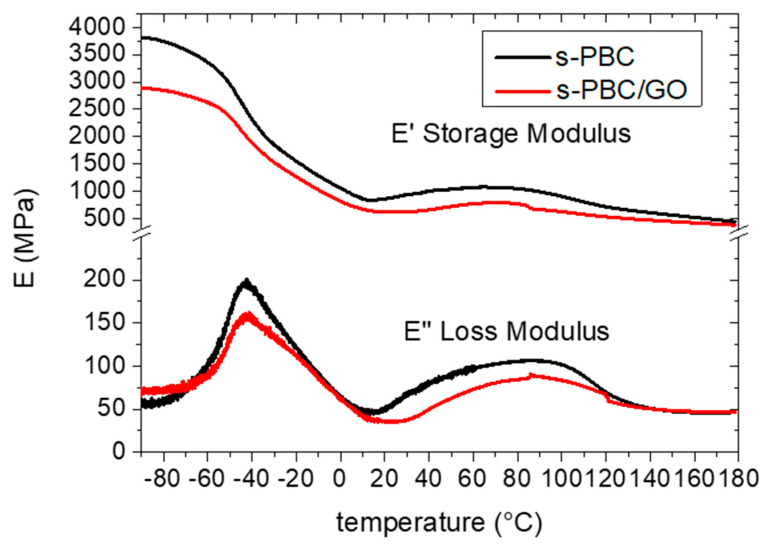
Dynamic mechanical analysis (DMA) curves for the storage and loss modulus for s-PBC and s-PBC/GO nanocomposite.

DMA was performed in order to investigate the viscoelastic properties of the polymer and the nanocomposite with GO [31]. The storage modulus E’ and loss modulus E″ are plotted as a function of temperature in Figure 4. The storage modulus is a measurement of the elastic behaviour of the material and thus its ability to bear a certain load and return to its initial shape once the load is removed. The ability of the material to withstand higher loads without being deformed in an irreversible way is as high as its E’ value. While E’ measures the stored energy, the energy dissipated as heat is represented by the viscous portion E”. For both the polymer and the nanocomposite, the E’ modulus is higher with respect to E’’ in all the investigated temperature range, suggesting an elastic behavior of the polymeric material. In particular, we can observe that the dispersion of GO inside the polymer increases the storage modulus by around 30%.

The glass transition temperature corresponds to the maximum in the E’’ curve or as a rapid decreasing of E’ value. Lower T_g_ values indicate a higher mobility of polymeric chains affecting the transport properties of the polymer. For the polymeric materials, two T_g_ values were observed and attributed to HI incompatibility with tBS, PS and sPS [31]. T_g_ values calculated using the E” curves are −42 °C and 86 °C, in agreement with data reported in [27]. The observed T_g_ values are the same for s-PBC and s-PBC/GO, showing that the dispersion of GO flakes increased the elastic properties of the polymer without affecting its T_g_. The lower T_g_ is attributed to hydrogenated isoprene and the second one to polystyrene and tert-butyl styrene blocks.

**Figure 5:**
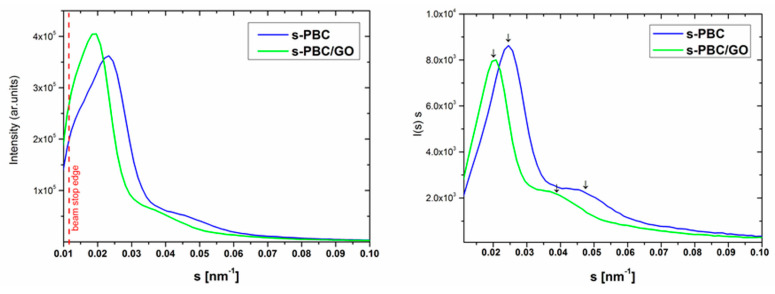
Small-angle X-ray scattering (SAXS) profiles (left side) and one-dimensional scattering curves (right side) for s-PBC and s-PBC/GO membranes.

One of the advantages of using Nexar™ polymer is the micellar morphology of the dilute pentablock copolymer solution that depends on the choice of the solvent (polar or a-polar), and determines the morphology of the cast films. The use of polar solvents leads to the formation of channels that are desirable for the application of this polymer in water purification as adsorbent or filter [18,23]. As was proved by Spontak et al. [32] in the case of T-EP-S/sS-EP-T pentablock copolymer, the supermolecular structure is formed by separated and randomly distributed spherical and/or ellipsoidal microdomains when low-polar solvent is used, such as cyclohexane or triisopropanolamine (TIPA). On the contrary, when high-polar tetrohydrofuran (THF) solvent was applied an ordered and complex morphology occurred, composed of domains with hexagonally-packed cylinders and domains with a lamellar superstructure. Moreover, by using different agents for staining samples investigated using the TEM method, it was established that corresponding to the lamellar superstructure a) the thickness of ion-rich lamellae reaches ca 12–14 nm and b) the thickness of nonpolar lamellae reaches ca 25–29 nm. This means that the so-called long period of that lamellar superstructure should amount to ca. 37–43 nm. We investigated the supermolecular structure of as obtained membranes using the SAXS method. The comparison of SAXS diffraction curves obtained as a result of measurements performed for s-PBC and s-PBC/GO samples are reported on the left side in Figure 5 in the suitable limited range of the scattering vector s = 0.01125 nm^−1^ ÷ 0.1000 nm^−1^. In both cases the broad and very strong scattering maximum appears in the range of the lowest scattering vector values and a smeared and weak scattering maximum is observed as a shoulder. It should be noticed that the strong diffraction maximum for s-PBC/GO lies closer to the beginning of the s axis than the strong diffraction maximum for s-PBC. For precise determination of the position of diffraction maxima, the diffraction curves have been transformed to the so-called one-dimensional scattering curves I(s)s vs. s. The curves are reported on the right side in Figure 5 and results concerning a) the s_m_ positions of maxima occurring on those curves and b) the d_B_ Bragg spacing (d_B_ = 1/s_m_) are presented in Table 1. Taking into account that polar DMF solvent was used for the preparation of the films and the results obtained by Mineart et al. [32], one can assume that the SAXS maxima detected for the s-PBC and s-PBC/GO cast films arise from x-ray scattering due to a lamellar superstructure occurring in these films. Consequently, the d_B_ spacing related to the positions of strong maxima observed in Figure 5 is the long period (L) of the lamellar superstructure. Thus, it is clear that the smeared weak maxima occurring on the SAXS curves in Figure 5 are the second order diffraction maxima for those lamellar superstructures, as is indicated by data of s_m_ given in Table 1 (the s_m_ position of the weak maximum is ca. twice bigger than the s_m_ position of the strong maximum). It is worth noting that the value of the long period determined in this work for the s-PBC membrane (L = 40.8 nm) fits well to the value of the long period deduced from data given in [32] (L = 37 ÷ 43 nm). Interestingly, the long period of the lamellar superstructure formed in the composed s-PBC/GO cast film (L = 51 nm) is significantly larger than the long period of the lamellar superstructure formed in the s-PBC cast film (L = 40.8 nm). This means that GO additives reside in lamellar stacks and most probably in the transition layer between ion-rich and nonpolar lamellae. Therefore, one can postulate that GO additives contribute to an increase of absorption sites density in the domains of the s-PBC/GO membrane with a lamellar superstructure as compared with absorption sites density in the domains of the s-PBC membrane with a lamellar superstructure.

### 3.2. Heavy Metals Removal

s-PBC and s-PBC/GO were tested for the removal of four metals (Ni, Co, Pb, Cr) from aqueous solutions containing metal salts (CoCl_2_, Ni(NO_3_)_2_, Cr_2_(SO_4_)_3_, Pb(NO_3_)_2_) at different concentrations. Table S1 in the supporting information reports, for each investigated metal salt, the amount of metal for the three salt concentrations used in this work: (a) 500 mg/L, (b) 1000 mg/L, (c) 2000 mg/L. These values were measured using EDXRF. The size/mass/charge values for each metal ion are reported in Table S2 of the supporting information. The investigated metal ions differ for their superficial charge and ion size: Ni^2+^ and Co^2+^ have a similar size and charge, Pb^2+^ has the same charge but a larger size and mass, Cr^3+^ has smaller dimensions and mass than Pb^2+^ but is comparable with Ni^2+^ and Co^2+^ and has the highest charge.

#### 3.2.1. Adsorption of Heavy Metals by s-PBC and s-PBC/GO

**Figure 6:**
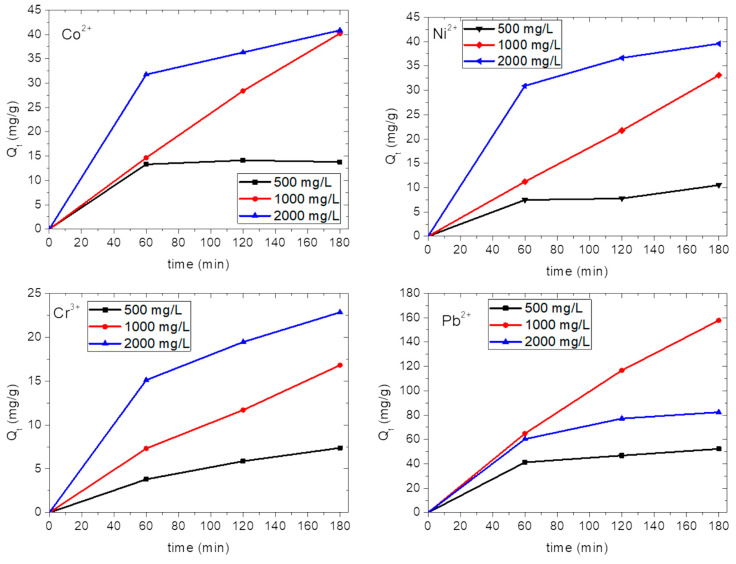
Adsorbed ions per gram of s-PBC(mg/g) versus contact time for different initial salt concentrations. Black, red and blue curves refer to 500, 1000 and 2000 mg/L, respectively.

Figure 6 shows the amount of metal ions adsorbed by s-PBC membranes, expressed as adsorbate/adsorbent weight ratio ~ Q_t_ (mg/g), as a function of contact time for all the salts at different initial concentrations. For all the initial salt concentrations, the adsorption affinity of s-PBC follows the trend Pb^2+^ > Co^2+^ > Ni^2+^ > Cr^3+^. For all the metal ions at the lower and the higher salt concentrations (black and blue curves, respectively) the adsorption occurs mainly in the first hour and then it slows down. At 500 mg/L, we observe a fast adsorption in the first hour and then Q_t_ remains constant with time, as evidenced by black curves. At 2000 mg/L, we observe a faster adsorption in the first hour, as evidenced by the slope of the blue curves in Figure 6; after 60 min, Q_t_ continues to increase more slowly. This means that in the first hour for the minimum concentration nearly the total amount of ions is removed, while for the maximum concentration the active sites on the polymeric membrane are almost saturated. At 1000 mg/L (i.e., red curves), the adsorption versus contact time shows approximately a linear increase for all the ions, at least for the entire time range (three hours) considered in this study.

**Figure 7:**
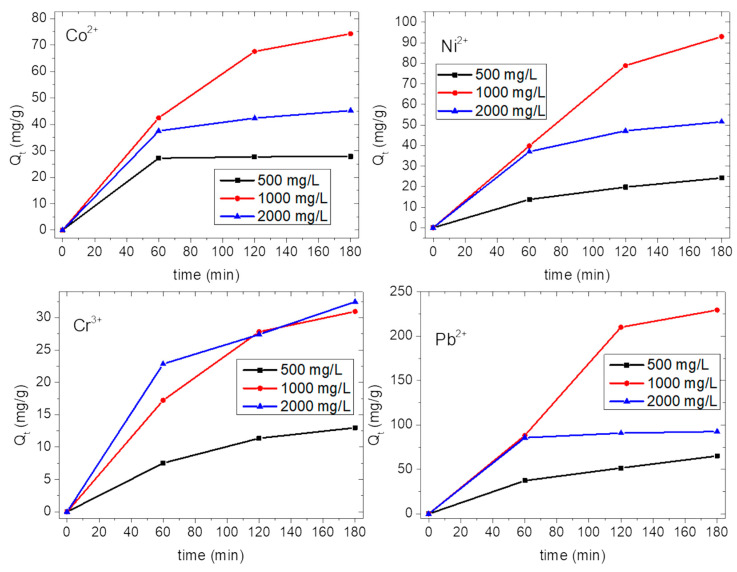
Amount of metal ions (Co^2+^, Ni^2+^, Cr^3+^, Pb^2+^) in mg adsorbed per gram of s-PBC/GO membrane in contact with the aqueous solutions, for different initial salt concentrations and as a function of time. Black, red and blue curves refer to 500, 1000 and 2000 mg/L, respectively.

Figure 7 shows the amount of ions adsorbed by s-PBC/GO (mg per gram of membrane) as a function of time during the immersion of the membrane in each metal salt solution, at different concentrations. As for s-PBC, at the lowest and the highest salt concentration (black and blue curves, respectively) the adsorption occurs mainly in the first hour and then it slows down. In particular, at 500 mg/L, we observe a fast adsorption in the first hour and then Q_t_ does not change significantly with time. At 1000 mg/L (i.e., red curves), the adsorption versus contact time has an increasing trend for all the ions in the first and second hour and then we observe a slight reduction of the adsorption rate between the second and the third hour.

At 2000 mg/L, we observe a fast adsorption in the first hour, as evidenced by the slope of the blue curves in Figure 7, and then the adsorption rate reduces for all the ions. As just explained for s-PBC, at the lowest and the highest concentrations, after one hour we observe the quasi total removal of ions and the saturation of active sites on the polymeric membrane, respectively.

As with s-PBC, s-PBC/GO also shows the highest adsorption capability for Pb^2+^ ions and the lowest for Cr^3+^ ions, independently of the initial salt concentration.

**Figure 8:**
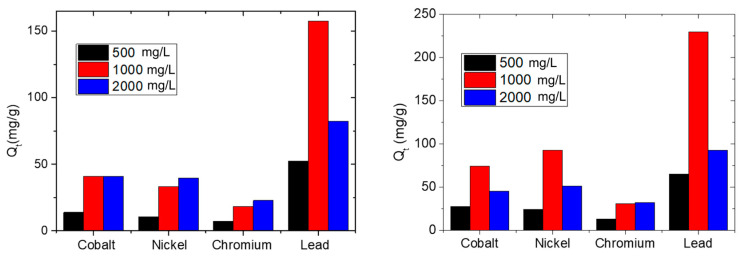
Adsorbed ions (mg) per gram of s-PBC (on the left) and s-PBC/GO (on the right) after 180 min of contact of the polymeric membrane with the aqueous solutions, for different initial salt concentrations.

Figure 8 reports the Q_t_ after three hours of contact of s-PBC and s-PBC/GO membranes with the metal salt solution, for all the ions at different initial salt concentrations, as indicated by black, red and blue bars for 500, 1000 and 2000 mg/L, respectively. The corresponding values are also reported in Table 2 and Table 3.

Figure 8 (on the left) and Table 2 show that after three hours of contact, the ions adsorbed by the s-PBC membrane (Q_t_) in a solution with 1000 mg/L salt concentration are about three times more than the ions adsorbed by the same membrane in a solution with 500 mg/L salt concentration. In particular, at the lowest concentration all the ions were adsorbed in the first hour, and then Q_t_ does not increase significantly, due to the depletion of ions from the solution (see Figure 6). The maximum adsorption of Co^2+^ ions by s-PBC is 41.1 mg/g after three hours at 1000 mg/L. At 2000 mg/L we do not observe a higher value of Q_t_. Ni^2+^ and Co^2+^ ions are very similar in terms of dimension, electronegativity and charge (see Table S2 in supporting information). Consequently, a similar trend with time is observed for Ni^2+^ ions (see Figure 6), and the highest final value of Ni^2+^ adsorption is 39.5 mg/g at 2000 mg/L. For all the concentrations, the lowest adsorption occurs for the trivalent ion Cr^3+^, and this is ascribed to the lower probability for a trivalent coordination to occur with sulfonic groups on the polymeric membranes; the maximum adsorption value is 22.8 mg/g at 2000 mg/L. The highest adsorption ability is reported for Pb^2+^ ions (157.7 mg/g after three hours at 1000 mg/L), underlying the higher affinity of Pb^2+^ with sulfonic groups. At the highest salt concentration, we observe that the final Q_t_ value is almost equal to or slightly higher than the ones measured at 1000 mg/L for all the ions, except for Pb^2+^. This confirms that at 2000 mg/L the adsorption occurs mainly in the first hour forced by the gradient of ions concentration and then is constant due to a passivation of active sites. In the case of Pb^2+^, the adsorption efficiency drops down for a salt concentration of 2000 mg/L with respect to 1000 mg/L. We can explain this behaviour considering that at the beginning Pb^2+^ adsorption occurs on the surface of the membrane and then it should diffuse inside the polymeric films. Pb^2+^ is the biggest and heaviest ion investigated in this work, and therefore, it is likely that at the highest concentration all the surface sites are covered fast, hindering further diffusion inside the polymer.

Figure 8 (on the right) also reports the Q_t_ values after three hours of contact with the s-PBC/GO membrane for all the metal ions at different initial salt concentrations, as indicated by black, red and blue bars for 500, 1000 and 2000 mg/L, respectively. The corresponding values are also reported in Table 3. The observed Q_t_ values are higher than the ones achieved by s-PBC membrane, confirming that the dispersion of a very low amount of GO inside the polymeric matrix increases its adsorption efficiency significantly. The s-PBC/GO membrane adsorbs more and faster (as evidenced by the slopes of the curves in Figure 6 and Figure 7) than the s-PBC membrane, independently of initial salt concentrations.

At 500 mg/L the adsorption affinity of s-PBC/GO follows the same trend as s-PBC, i.e., Pb^2+^ > Co^2+^ > Ni^2+^ > Cr^3+^. At 1000 and 2000 mg/L, the affinity for Ni^2+^ is higher than for Co^2+^ in the case of composite membrane, and the observed trend is Pb^2+^ > Ni^2+^ > Co^2+^ > Cr^3+^. Considering that for the composite membranes the active sites for adsorption can be both the sulfonic groups of the polymeric matrix and the oxygen functionalities on the GO layers, the observed results indicate a higher affinity of Ni^2+^ with respect to Co^2+^ for GO sheets. However, this aspect should be further investigated.

As for the s-PBC membrane, the final Q_t_ value after three hours of contact with the membrane increases by about three/four times by increasing the initial salt concentration from 500 to 1000 mg/L. On the contrary, at 2000 mg/L the final Q_t_ is slightly higher than the ones measured at 1000 mg/L only for Cr^3+^ ions (for s-PBC the slight increase was for both Ni^2+^ and Cr^3+^), while for all the other metals Q_t_ decreases. As for s-PBC, the Q_t_ measured for all the ions at 2000 mg/L is higher than the ones at 500 mg/L; therefore, for the lowest concentration Q_t_ does not increase after one hour due to the depletion of ions in the solution, while for the highest concentration, a very fast adsorption takes place due to the larger Pb^2+^ ions concentration gradient, causing the passivation of active sites on the membrane surface. The formation of a blocking layer prevents further ions adsorption. This is not observed with Cr^3+^ ions since its adsorption by s-PBC/GO is lower than for the other ions.

Comparing Table 2 and Table 3, we observe a higher adsorption efficiency of the composite with respect to the polymer itself for all the ions: the maximum adsorption of Co^2+^ ions by s-PBC/GO is 74.2 mg/g at 1000 mg/L (1.8 times higher than for s-PBC, i.e., 41.1 mg/g); the highest final value of Ni^2+^ adsorption is 93 mg/g at 1000 mg/L (2.3 times higher than s-PBC, i.e., 39.5 mg/g); the maximum adsorption value of Cr^3+^ is 32.5 mg/g at 2000 mg/L (22.8 mg/g for s-PBC). As observed in the case of s-PBC, for Pb^2+^ ions, at the lowest and highest concentrations, the adsorption occurs in the first hour and then it does not change significantly. At 1000 mg/L, the adsorption increases with contact time, and after three hours the maximum adsorption is observed with Q_t_ = 229.4 mg/g (157.7 mg/g for s-PBC). These values confirm the higher affinity of Pb^2+^ ions with sulfonic groups and oxygen functionalities of GO layers with respect to other metals. At 2000 mg/L, as for other metallic ions, except for Cr^3+^, which has the lowest adsorption, we observe a reduction of the value of Q_t_. As explained above, this is due to the fast adsorption occurring on the surface of the membrane and preventing the ions from diffusing inside the polymeric films.

**Figure 9:**
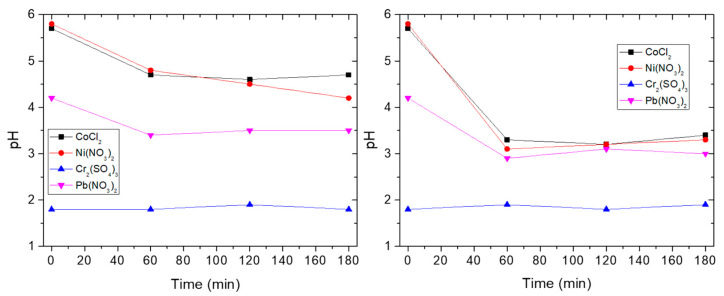
pH values of metal salt solutions measured as a function of contact time with the s-PBC (on the left) and s-PBC/GO (on the right) membrane.

In order to better understand the adsorption mechanisms, we measured the pH of the solutions before and after immersion of the s-PBC and s-PBC/GO membranes. The pH values measured during the immersion time of the membrane in salt solutions at 2000 mg/L are reported in Figure 9.

For the solution containing Cr^3+^, the pH is already below 2 before membrane immersion and remains almost constant, therefore, it is not possible to distinguish an effect of the membrane on the pH of the solution; for all the other ions (Pb^2+^, Co^2+^ and Ni^2+^) the pH changes mainly in the first hour and then it remains almost constant. The decreasing of pH is simultaneous with the adsorption of metallic ions in the first hour. This is a further proof that the adsorption is due to the interaction of metal ions with the sulfonic groups of the membrane. We observed the same trend for the salt solutions in contact with s-PBC/GO membrane. In particular, the decreasing of pH values in the first hour is higher for s-PBC/GO with respect to s-PBC; similarly, the adsorption abilities shown by s-PBC/GO were higher with respect to s-PBC.

#### 3.2.2. Heavy Metals Removal: A Kinetic Study

The kinetic analysis is useful from a practical point of view to determine the time required to complete the adsorption process and to understand the mechanisms underlying the removal processes. For this purpose, we consider the experiments performed at an initial salt concentration of 1000 mg/L, since at this value the adsorption of metal ions increases with contact time for both the s-PBC and s-PBC/GO membranes and for all the investigated ions, as evidenced by Figure 6 and Figure 9. At 500 and 2000 mg/L, we observed a condition of equilibrium or pseudo equilibrium, which are not appropriate for kinetic studies (see Figure 6 and Figure 7).

Different kinetic models can be used for studying the adsorption phenomenon, and fitting the experimental data with the models permits elucidation of the adsorption mechanism.

Generally speaking, there are three steps in an adsorption process: (i) film diffusion, which is the external mass transfer of the adsorbate from the bulk solution to the external surface of the adsorbent, (ii) pore diffusion or intraparticle diffusion, which is the transport of adsorbate particles from the external surface into the pores of the adsorbent medium and (iii) the sorption itself, i.e., the surface reaction to attach the adsorbate particles to the internal surface of the sorbent [33,34]. The kinetic models are divided in two main categories, according to the rate limiting step in the adsorption process: some models are based on the fact that the sorption is the rate limiting step (adsorption reaction models) and these are usually suitable to explain chemisorption; others suppose that the diffusion is the rate limiting step (adsorption diffusion models) and these are well suited with physisorption processes [35]. Pseudo-first-order (PFO) or pseudo-second-order (PSO) and intra-particle (IP) models are some examples of adsorption reaction models and diffusion reaction models, respectively. In order to study adsorption kinetics, the linear forms are applied and the suitability of any model depends on the degree of linear correlation between the experimental and the predicted values (R^2^) [34].

The PFO and PSO models rest on the main assumption that adsorption only occurs on localized sites and involves no interaction between the adsorbed ions [35]. According to these models, the process rate depends on the availability of adsorption sites on the surface of the adsorbent rather than on the adsorbate concentration in the bulk solution [36].

Also known as the Lagergren model [36], PFO describes the adsorption of solute onto adsorbent following the first order mechanism. The linear expression of PFO is reported in Equation (1), the value of k is determined by plotting ln(Q_e_ − Q_t_) versus t.

(1) $$\ln\left(Q_{e}-Q_{t}\right)={\mathrm{lnQ}}_{e}-k_{1}t$$

where Q_t_ is the amount of adsorbate adsorbed onto the adsorbent at time t (mg/g), Q_e_ is the equilibrium adsorption capacity (mg/g), and k_1_ is the rate constant (min^−1^).

In recent years, the pseudo-second-order rate expression has been widely applied to the adsorption of pollutants from aqueous solutions in order to describe chemisorption involving covalent forces and ion exchange between the adsorbent and adsorbate [36]. The linear expression of PSO is reported in Equation (2); the value of k is determined by plotting t/Q_t_ versus t.

(2) $$\frac{t}{Q_{t}}=\frac{1}{K_{2}Q_{e}{}^{2}}+\frac{t}{Q_{e}}$$

where Q_t_ is the amount of adsorbate adsorbed onto the adsorbent at time t (mg/g), Q_e_ is the equilibrium adsorption capacity (mg/g), and k_2_ is the rate constant per min.

In the following paragraphs, the kinetic studies of heavy metals adsorption by s-PBC and s-PBC/GO are reported only for the results obtained at 1000 mg/L as initial salt concentration, for the reasons explained at the beginning of this section. We have chosen to use the pseudo-second-order kinetic model and the intraparticle diffusion model. With respect to the first order model, the advantage of using the second order model is that the equilibrium capacity can be directly calculated from the model. Furthermore, in most cases, the PFO model is inferior in terms of fit to the PSO model using a least-square discrimination procedure [33]. The wide applicability of PSO over PFO was shown by Plazinski et al. to be dependent on mathematical basis rather than physical basis [37].

Both PSO and PFO do not explain the diffusion of solute into the adsorbent; therefore, diffusion models should be investigated [38]. Most studies have shown that the mass transfer is the rate-controlling parameter for the first few minutes of the adsorption process and that the parameter that controls the process for most of the contact time is intraparticle diffusion [39]. The adsorption of solute from a solution into a sorbent involves mass transfer of adsorbate (film diffusion), surface diffusion and pore diffusion. Film diffusion is an independent step, whereas surface and pore diffusion may occur simultaneously. The intraparticle diffusion equation (Equation (3)), as described by Weber and Morris [39], may be applied in the determination of the intraparticle diffusion rate constant, k_d_, and the boundary resistance, C.

(3) $$Q_{t}=k_{d}\left(t^{0.5}\right)+C$$

where Q_t_ is the amount of adsorbate adsorbed onto the adsorbent at time t (mg/g), k_d_ is the rate constant (mg/g)min^0.5^, and C determines the boundary layer effect (i.e., higher values, larger film diffusion resistance) and is linked to external mass transfer [34]. Using the plot of Q_t_ versus t^0.5^, it is possible to determine k_d_ and C. When intraparticle diffusion alone is the rate limiting step, then the plot of Q_t_ versus t^0.5^ passes through the origin (C = 0). When film diffusion also takes place then the intercept is C > 0, which gives an idea of the thickness of the boundary layer [33]: the larger the intercept the greater the boundary layer effect, i.e., the film resistance to mass transfer surrounding the adsorbent is greater [40]. Generally speaking, the faster the adsorption of ions is the higher the probability of a layer of ions being formed on the adsorbent surface (boundary layer on the membrane surface) is [34].

Table 4 reports R^2^ values obtained by fitting the experimental data for heavy metal ions removal by s-PBC and s-PBC/GO with the pseudo-second-order kinetic model and intraparticle diffusion model, respectively.

For all the investigated materials the higher R^2^ values are obtained by fitting the data with the intraparticle diffusion model, confirming that the parameter controlling the process for most of the contact time is the intraparticle diffusion. As a consequence, we discuss in the following paragraph the obtained results for s-PBC and s-PBC/GO according to this model. The related fitting parameters are reported in Table 5.

In the intraparticle diffusion model [39], the slope corresponds to the kinetic constant for the adsorption mechanism expressed in mg/(g×min^0.5^) for each metal. The intercept C (mg/g) (not shown in the table) determines the boundary layer effect. The calculated values of the C parameter for s-PBC are (−20.4 ± 1.6) mg/g for Co^2+^, (−19.0 ± 4.2) mg/g for Ni^2+^, (−5.8 ± 2.2) mg/g for Cr^3+^ and (−62.2 ± 1.6) mg/g for Pb^2+^. The values calculated for s-PBC/GO are close to zero within errors independently of the investigated metals. These values indicate that diffusion is the main mechanism explaining the adsorption of heavy metals by s-PBC and s-PBC/GO nanocomposite.

Considering the data reported in Table 2 and Table 3 for the different metal ions, Q_t_ values for s-PBC and s-PBC/GO follow the same trend of the diffusion rate constant k_d_ (i.e., Pb^2+^ > Co^2+^ > Ni^2+^ > Cr^3+^ for s-PBC and Pb^2+^ > Ni^2+^ > Co^2+^ > Cr^3+^ for s-PBC/GO, respectively). The increased adsorption efficiency of the nanocomposite is also shown by the increasing values of the k_d_ parameter for all the investigated ions using s-PBC/GO with respect to s-PBC.

Pb^2+^ is the ion adsorbed in larger amounts and at a higher rate, confirming the high affinity of Pb^2+^ with sulfonic groups on the polymer and also with GO functionalities in the case of the nanocomposite.

Co^2+^ and Ni^2+^ are similar in terms of electronegativity and dimension (see Table S2 of supporting information), and they showed the same affinity for s-PBC as reported in the previous paragraphs. Co^2+^ and Ni^2+^ are adsorbed by coordination with two sulfonic groups, as for Pb^2+^ ions, and thus their adsorption is favoured with respect to Cr^3+^, either in terms of Q_t_ or the rate constant. The dispersion of GO inside the polymeric matrix enhanced the adsorption of both ions, in particular for Ni^2+^. The slightly higher affinity of s-PBC/GO for Ni^2+^ with respect to Co^2+^ is also confirmed by kinetic data, i.e., the constant k_d_ for Ni^2+^ is higher than for Co^2+^. On the contrary, Cr^3+^ is the least adsorbed ion by s-PBC/GO since, as just explained for s-PBC, its adsorption occurs via coordination with three anionic groups and this is not favoured by steric hindrance.

A direct comparison among different polymeric materials cannot be performed easily, since the adsorption capacity of the materials depends on the amount of active sites and the experimental conditions, such as weight ratio between adsorbent and metal ions to be adsorbed from a solution, pH and temperature of the solution, nature of counter ion, etc. However, just to have a comparison with other results reported in the literature, we observe that for Nafion [20], another sulfonated polymer, the adsorption efficiency of Pb^2+^ ions in terms of Q_t_ is lower by about 3 times than for s-PBC. This can be ascribed to the higher number of active sites (i.e., sulfonic groups), underlying their high affinity with Pb^2+^ ions.

The s-PBC/GO nanocomposite also showed higher or at least similar adsorption efficiency with respect to the use of other graphene-based materials reported in the literature. By using a small amount of GO dispersed in s-PBC (0.003 %wt of GO with respect to s-PBC) we achieved a Q_t_ value of 74.2 mg/g for Co^2+^, compared, for example, with the value of 68.2 mg/g for Co(II) removal on graphene oxide nanosheets reported by Zhao et al. [13]. Concerning Ni^2+^ ions, we obtained an adsorption efficiency of 93 mg/g by using the same small amount of GO, compared with a value (reported in the literature) of 114.4 mg/g obtained by using magnetically recoverable graphene/Fe_3_O_4_ composite [41]. S-PBC/GO showed the best removal efficiency for Pb^2+^ ions: we observed a Q_t_ value of 229.4 mg/g in our experimental conditions, and this result is higher than most of the values we found in the literature. Just to give some comparisons, the adsorption capacity for Pb^2^^+^ was estimated to be 76.94 mg/g for magnetic chitosan grafted with GO sheets [42], 100 mg/g for MnFe_2_O_4_^-^ graphene composite [43] and 166.66 mg/g using carbon nanofiber grown on powered activated carbon [44].

## 4. Conclusions

In this work, s-PBC and s-PBC/GO nanocomposite membranes were investigated for the first time as adsorbents for the removal of heavy metal ions from aqueous solutions. The membranes were prepared using the solvent casting method with DMF polar solvent. The use of polar solvent allowed s-PBC membrane to be obtained with a complex morphology consisting of domains with a lamellar superstructure characterized by a long period of ca. 41 nm. The dispersion of GO inside the polymer matrix maintains the lamellar superstructure but increases the spacing between lamellae (up to 51 nm), forming a more porous structure that favours the adsorption ability of the polymer itself.

s-PBC has shown good efficiency in heavy metal ions removal in the following order Pb^2+^ > Co^2+^ > Ni^2+^ > Cr^3+^, due to the presence of sulfonic groups that play a fundamental role in the adsorption process of metal ions. The adsorption efficiency of this polymer in terms of Q_t_ and in terms of kinetic rate constant was highly increased by the dispersion of a low amount of GO. In particular, the presence of GO inside the polymeric matrix increases the affinity of the adsorbent for Ni^2+^ with respect to Co^2+^, and hence, for s-PBC/GO the affinity for the heavy metals and the removal velocity follow the order Pb^2+^ > Ni^2+^ > Co^2+^ > Cr^3+^. As a consequence of the dispersion of GO inside the polymeric matrix, for Co^2+^, Cr^3+^, Pb^2+^ and Ni^2+^ the Q_t_ value increases by 1.8, 1.7, 1.5 and 2.8 times, with respect to the those measured for the polymer itself.

From the kinetic study we found that for all the investigated materials the parameter controlling the process for most of the contact time is intraparticle diffusion. Diffusion is the main mechanism explaining the adsorption of heavy metals by both s-PBC and s-PBC/GO nanocomposite membranes. The kinetic parameters obtained by modelling the adsorption process indicate that for s-PBC/GO the removal rate of the metal ions is higher than for s-PBC. This could be ascribed to the morphological changes introduced by GO in terms of high porosity, high roughness and longer lamellar distances, along with a higher density of adsorption sites (sulfonic and oxygen functionalities) available for the ions.

In conclusion, s-PBC has been shown to be an efficient and cheap polymer for the preparation of membranes to be applied in heavy metals removal. The dispersion of a low amount of GO in a s-PBC matrix highly increased the removal performance of the polymer, with adsorption capacities that are higher than the ones achievable with the use of GO flakes. In addition to this, the use of polymeric nanocomposite has the advantage of avoiding the all problems linked to the dispersion of nanomaterials in the environment when using adsorbent media in the form of powders directly dispersed in water. Another advantage could be the possibility to recover metal ions from the adsorbent membranes after the water purification process. In our opinion, in the future this kind of material could allow two environmental issues to be tackled simultaneously: the removal of contaminants and their recovery and re-use.

## Figures and Tables

**Table 1:** The s_m_ diffraction maxima positions and the d_B_ Bragg spacings.

Membranes	s_m_ [nm^−1^]		d_B_ [nm]
s-PBC	0.0245	0.0478	40.8
s-PBC/GO	0.0196	0.0382	51.0

**Table 2:** Amount (mg) of metal ions adsorbed per gram of s-PBC membrane, expressed as mg/g, after 180 min immersion of the membrane in the metal salt solutions, for different initial concentrations.

Metal ion	Q_t_ @ 500 mg/L	Q_t_ @ 1000 mg/L	Q_t_ @ 2000 mg/L
Co^2+^	13.8	41.1	40.8
Ni^2+^	10.5	33.0	39.5
Cr^3+^	7.4	18.3	22.8
Pb^2+^	52.4	157.7	82.4

**Table 3:** Amount of metal ions adsorbed per gram of s-PBC/GO membrane, expressed as mg/g, after 180 min immersion of the membrane in the metal salt solutions, for different initial concentrations.

Metal ion	Q_t_ @ 500 mg/L	Q_t_ @ 1000 mg/L	Q_t_ @ 2000 mg/L
Co^2+^	27.8	74.2	45.2
Ni^2+^	24.2	93.0	51.6
Cr^3+^	13.0	30.9	32.5
Pb^2+^	65.2	229.4	92.7

**Table 4:** R^2^ values obtained by fitting the experimental data for metal ions removal at 1000 mg/L by s-PBC and s-PBC/GO, using the pseudo-second-order kinetic model and the intraparticle diffusion model.

Metal	Pseudo Second Order t/Q_t_ = (1/Q_e_)t + (1/kQ_e_^2^)		Intraparticle Diffusion Model Q_t_ = k_d_t^0.5^ + C	
	s-PBC	s-PBC/GO	s-PBC	s-PBC/GO
Co^2+^	0.93234	0.9478	0.998	0.9851
Ni^2+^	0.44909	0.55041	0.98042	0.95149
Cr^3+^	0.75785	0.94692	0.97122	0.98622
Pb^2+^	0.99878	-0.51716	0.99983	0.90144

**Table 5:** Q_t_ values and kinetic constants calculated according to the intraparticle diffusion model for the removal of metal ions by s-PBC and s-PBC/GO at 1000 mg/L.

s-PBC			s-PBC/GO	
**Metal**	Q_t_ (mg/g) After 180 min	k_d_ (mg/(g×min^0.5^))	Q_t_ (mg/g) After 180 min	k_d_ (mg/(g×min^0.5^))
**Co^2+^**	41.1	4.5 ± 0.1	74.2	5.7 ± 0.4
**Ni^2+^**	33.0	3.8 ± 0.4	93.0	7.0 ± 0.9
**Cr^3+^**	18.3	1.7 ± 0.2	30.9	2.4 ± 0.2
**Pb^2+^**	157.7	16.4 ± 0.1	229.4	17.9 ± 3.3

## Supplementary Materials

The following are available online at https://www.mdpi.com/2079-4991/10/6/1157/s1, Figure S1: FT-IR spectrum of GO; Table S1: Amount of metal ions in mg/L at different salts concentrations, i.e., 500 mg/L (a), 1000 mg/L (b), 2000 mg/L (c); Table S2: Values of ionic radii, electronegativity and atomic mass for the investigated cations.
