# Supplementary material for: Sulfonated Pentablock Copolymer Membranes and Graphene Oxide Addition for Efficient Removal of Metal Ions from Water
Source: Nanomaterials (Basel). 2020 Jun 12;10(6):1157. doi: 10.3390/nano10061157 (PMC7353454; doi:10.3390/nano10061157)
Supplement: Supplementary file 1 [file nanomaterials-10-01157-s001.pdf]

Sulfonated Pentablock Copolymer Membranes and Graphene Oxide Addition for Efficient Removal of Metal Ions from Water

Simona Filice [1,*], Marta Mazurkiewicz-Pawlicka [2], Artur Malolepszy [2], Leszek Stobinski [2,3], Ryszard Kwiatkowski [4], Anna Boczkowska [5], Leon Gradon [2] and Silvia Scalese [1,*]

1 Istituto per la Microelettronica e Microsistemi, Consiglio Nazionale delle Ricerche (CNR-IMM), Ottava Strada n.5, I-95121 Catania, Italy
2 Faculty of Chemical and Process Engineering, Warsaw University of Technology, ul. Warynskiego 1, 00–645 Warsaw, Poland; Marta.pawlicka@pw.edu.pl (M.M-P.); artur.malolepszy@pw.edu.pl (A.M.); lstob50@hotmail.com (A.M.)
3 NANOMATERIALS LS, Wyszogrodzka 14/38, 03–337 Warsaw, Poland
4 Institute of Textile Engineering and Polymer Materials, University of Bielsko-Biała, Willowa 2, 43-309 Bielsko-Biała Poland; rkwiatkowski@ath.bielsko.pl (R.K.)
5 Faculty of Materials Science and Engineering, Warsaw University of Technology, ul. Woloska 141, 02–507, Warsaw, Poland; anna.boczkowska@pw.edu.pl (A.B.); leon.gradon@pw.edu.pl (L.G.)
* Correspondence: Simona.Filice@imm.cnr.it (S.F.); silvia.scalese@imm.cnr.it (S.S.)

Supporting Information

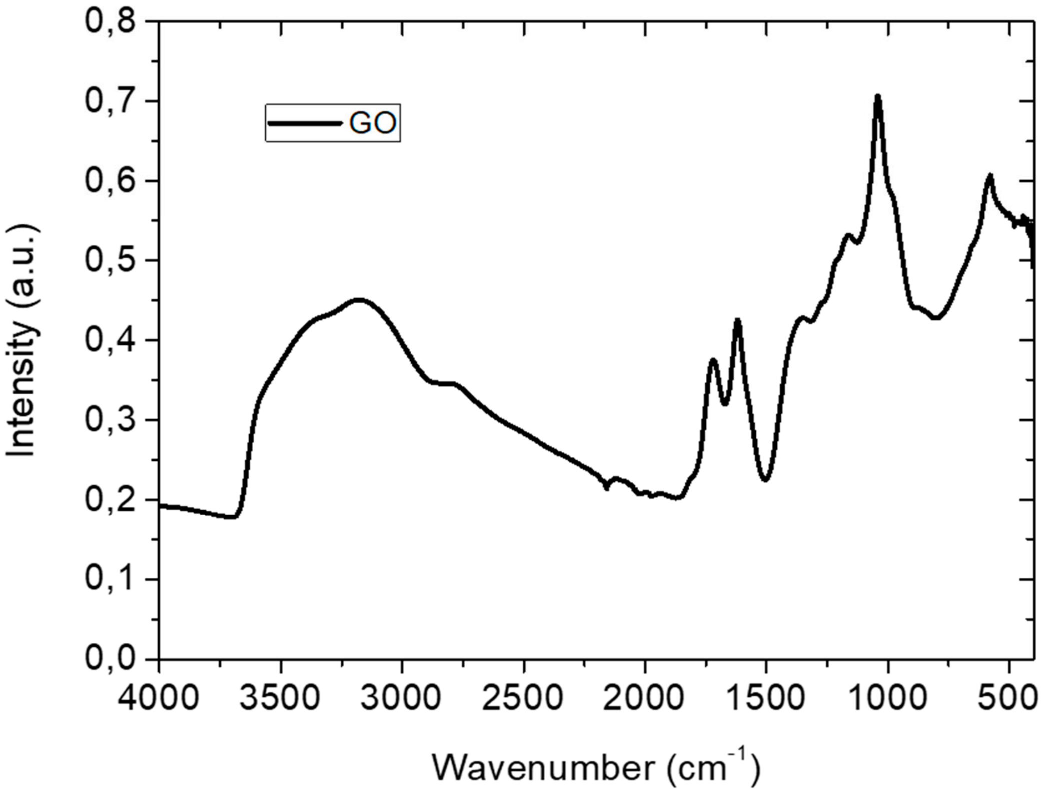

Figure 1. *FT-IR spectrum of GO.*

Table 1. Amount of each metals in mg/L at different salts concentrations i.e. 500 mg/L (a), 1000 mg/L (b), 2000 mg/L (c).

Salt	Metal (%)	Metal Concentration (mg/L)[(a)]	Metal Concentration (mg/L)[(b)]	Metal Concentration (mg/L)[(c)]
**$CoCl_2$**	25	118.3	261.9	502
**$Ni(NO_3)_2$**	20	102	221.2	435.8
**$Cr_2(SO_4)_3$**	14.5	75	131.6	257.9
**$Pb(NO_3)_2$**	62.5	308.5	698.5	1040

Table 2. Values* of ionic radii, electronegativity and atomic mass for the investigated cations.

	Ionic radii (amstrong) [ref.]	Electronegativity (Pauling)	Atomic Mass (u.a.)
**Cr^{3+}**	0.615	1.66	51.9961
**Co^{2+}**	0.58-0.9	1.88	58.9332
**Ni^{2+}**	0.49-0.69	1.91	58.6934
**Pb^{2+}**	0.98-1.48	2.33	207.2

* *"Revised Effective Ionic Radii and Systematic Studies of Interatomic Distances in Halides and Chalcogenides"* By **R. D. Shannon**. Central Research and Development Department, Experimental Station, E. I. Du Pont de Nemours and Company, Wilmington, Delaware 19898, U.S.A. Published in Acta Crystallographica. (1976). A32, Pages 751-767.
